# Case report: From anxiety disorders to psychosis, a continuum in transitional age youth?

**DOI:** 10.3389/fpsyt.2022.990138

**Published:** 2022-10-03

**Authors:** Joana Reis, Simone Marchini, Hélène Nicolis, Véronique Delvenne

**Affiliations:** ^1^Faculty of Medicine, Université Libre de Bruxelles, Brussels, Belgium; ^2^Child and Adolescent Psychiatry Department, Queen Fabiola Children's University Hospital, Brussels, Belgium; ^3^Child and Adolescent Team, Mental Health Service at Université Libre de Bruxelles, Brussels, Belgium; ^4^Child and Adolescent Psychiatry Department, Erasme Hospital, Brussels, Belgium

**Keywords:** anxiety disorders (AD), ultra-high risk (UHR), psychosis, schizophrenia—spectrum disorders, transitional age youth, case report

## Abstract

**Introduction:**

To date, among individuals meeting ultra-high risk criteria for psychosis, the relationship between the presence of anxiety disorders and the risk of psychotic transition raises several unanswered questions.

**Case description:**

This case report describes the clinical progression of a 17-year-old male initially presenting anxious symptoms meeting the DSM-V criteria for panic disorder. The patient also reported social withdraw, mild depressive symptoms, insomnia and fatigue. Over a 6 month period, a gradual onset of subthreshold psychotic symptoms suggested a prodromal phase of a psychotic disorder.

**Diagnostic assessment and therapeutic intervention:**

A detailed assessment of UHR criteria for psychosis was performed. The overall level of social and occupational functioning was assessed by the SOFAS, which showed a 35% drop over a 12 months period. The CAARMS, has also been administered. The patient met the diagnostic criteria for UHR, APS group. The care plan included psychiatric follow-up, pharmacologic treatment, individual psychological follow-up and individual and familial psychoeducation. Over a 6 months period, the patient did not experienced a first psychotic episode and presented a partial improvement of psychotic symptoms.

**Conclusion:**

The DSM-V categorical approach does not seem to adapt well to early clinical presentations in transitional age youth. A transdiagnostic and dimensional approach allows to better identify at-risk patients of psychiatric disorders and implement early intervention strategies.

## Introduction

As the onset of most psychiatric disorders typically occurs during late adolescence and early adulthood, transitional age youth (TAY) are an at-risk population in terms of mental health (1). Early intervention in mental health is crucial, as it seems promising in modifying long-term outcomes and reducing illness severity (2).

Recent research recognizes that current categorical frameworks for classification and treatment in psychiatry are inadequate, particularly in TAY. Trans-diagnostic clinical staging models have gained prominence, by allowing a multidimensional assessment and taking into account a continuum of illness (3).

Categorical diagnosis such as anxiety and schizophrenia have been considered as completely distinct entities for years, even if the comorbidity between them has long been recognized. Regarding schizophrenia, the initial diagnosis frequently occurs at the time of the first psychotic episode. However, the diagnosis is often preceded by a prodromal phase where several symptoms gradually emerge. Early symptoms may be non-specific and include anxiety, as well as depressed mood, social withdrawal and academic difficulties. Non-specific symptoms may be followed by the basic symptoms, subtle subclinical disturbances in cognition, perception, language, emotional reactivity and stress tolerance. Later, these abnormalities become more pronounced and subthreshold positive symptoms of psychosis also emerge (4). The broad range of symptoms present in these early stages of schizophrenia include a wide variety of anxiety symptoms and comorbid entities are often present (5). Thus, the identification of anxiety symptoms seems to be an essential step in the assessment of a potential prodromal phase, in particular, when evaluating a patient also presenting impaired cognitive or social functioning. Recent epidemiological studies also show that anxiety disorders, such as social anxiety disorder, panic disorder and obsessive-compulsive disorder, are more common among people diagnosed with psychotic disorders compared to the general population (6).

Current research establishes a new paradigm for schizophrenia prodrome, which is currently considered a flexible entity where symptoms can completely disappear, persist or progress in several possible directions. The term ultra-high risk (UHR) for psychosis has therefore been used to designate individuals who potentially present prodromal symptoms and may benefit from early intervention strategies (7). Most evidence-based recommendations for UHR point out cognitive-behavioral therapy (CBT) as the most efficacious intervention, improving social functioning, allowing reduction of psychotic symptomatology and preventing or delaying transition to psychosis. Studies on the benefit/ risk balance of antipsychotic medication were not conclusive and existing clinical guidelines do not recommend systematic antipsychotic use (8, 9).

To date, among UHR individuals, the relationship between the onset of anxiety symptoms and the risk of psychotic transition raises several unanswered questions and remains a topic of scientific interest. This case report presents the clinical situation of a 17-year-old adolescent complaining with anxious symptoms meeting the DSM-V criteria for panic disorder [300.01 (F41.0)]. The progressive emergence of prodromal symptoms, possibly suggesting a psychotic disorder, led to reflexions about anxiety disorders and psychosis as comorbid conditions or manifestations of the same clinical entity. The authors propose to include some considerations about the clinical and epistemological complexity of these categorical diagnoses. A trans-diagnostic dimensional approach is preferable in order to comprehensively assess the ever-changing clinical presentation and to provide appropriate care.

## Case description

We describe the clinical case of a white Caucasian 17-year-old male adolescent referred to an outpatient child and adolescent mental health service in the Brussels urban area, Belgium, after an initial psychological assessment in a private outpatient clinic. At the time of the first psychiatric assessment, the patient was the main requester of the consultation. He presented, for about 3 months, panic attacks, characterized mainly by trembling, palpitations, a sensation of shortness of breath and a fear of losing control, without a trigger factor. These episodes, which had increased in frequency and intensity since their onset, were accompanied by a persistent worry of further panic attacks and an increase in social withdrawal that had been gradually increase in intensity over about 3 years. This posture described by the patient as voluntary self-isolation and avoidance of interactions with peers and family, was not associated with fear or anxiety related to social interactions. The patient also reported mild depressive symptoms, a sleep disorder characterized by initial insomnia, and fatigue.

Early childhood development was described as normal by parents and there was no history of perinatal complications. The patient received speech therapy, from age 3 to, for speech delay (first words at 24-months old) and articulation disorders. He took his first steps at around 12 months of age. Social interactions with peers were spontaneous during childhood and early adolescence. Regarding scholar functioning, no learning difficulties had ever been reported and he was attending, at the time of the first consultation, the last year of secondary school.

Prior to the onset of symptoms, there were no known family stressors or any significant life events, apart from the emigration of the family, from another European country, 7 years later, for professional reasons. Family psychiatric history was also not relevant.

His medical history included a primary spontaneous pneumothorax at the age of 16. He was admitted to hospital for 1 week and a chest tube was inserted, without complications. Physical examination (including cardiovascular screening) was normal, with a body mass index of 19 kg/m^2^. All laboratory results (blood and urine tests) were within normal limits. There was no history of alcohol, tobacco or drug use and he had not been on any medications.

Intelligence quotient (IQ) was assessed using the Wechsler Intelligence Scale for Adults, 4^th^ Edition (10) and revealed a very superior IQ (140, percentile >99).

At the time of the first psychiatric assessment, Beck Depression Inventory II (BDI-II), was administered to measure the severity of depressive symptoms (11, 12). The patient scored for “mild depression” (score: 19). The Panic Disorder Severity Scale (PDSS), was also administered (13, 14). This self-report scale, assessing the severity of panic attacks and panic disorder symptoms, revealed a moderate intensity of symptoms (score: 12). Initial assessment outcomes are described in Table 1. The patient met the Diagnostic and Statistical Manual of Mental Disorders, 5th edition (DSM-V) criteria for panic disorder [300.01 (F41.0)] (15).

**Table 1:** Initial assessment results.

**Tools and subtest(s)**	**Score**	**Interpretation**
**WAIS-IV**		**Percentile**
IQ	140	>99
VCI	122	93
PRI	134	99
WMI	137	99
PSI	145	>99
**BDI-II**	19	Mild depression
**PDSS**	12	Moderately ill

BDI-II, Beck Depression Inventory, second version; IQ, Intelligence Quotient; PDSS, Panic Disorder Severity Scale; PRI, Perceptual Reasoning Index; PSI, Processing Speed Index; VCI, Verbal Comprehension Index; WAIS-IV, Wechsler Adult Intelligence Scale, fourth edition; WMI, Working Memory Index.

Pharmacologic and psychotherapeutic treatment options were discussed with the patient and parents (16). Since the patient was not motivated to start CBT, a selective serotonergic reuptake inhibitor, Sertraline 50 mg per day, was initiated, with a substantial improvement of anxiety and depressive symptoms, over a 3 months period. No unexpected side effects were described.

Approximately 6 months after starting antidepressant treatment, parents reported a more pronounced decrease in social functioning, despite continued pharmacologic treatment and complete remission of panic attacks. In particular, parents observed a global decrease in social interactions, including family interactions, and the onset of clinophilia. The patient additionally reported aboulia and concentration problems related to school but only a slight drop in school results was observed at this stage. Besides, the patient reported, for the first time, having, for about 4 months, subthreshold psychotic symptoms, such as suspiciousness ideas, visual distortions, and subjective changes in speech, such as thought blockage and intrusive thoughts. These recent symptoms caused a significant distress and fear to “go crazy”.

**Figure 1:**
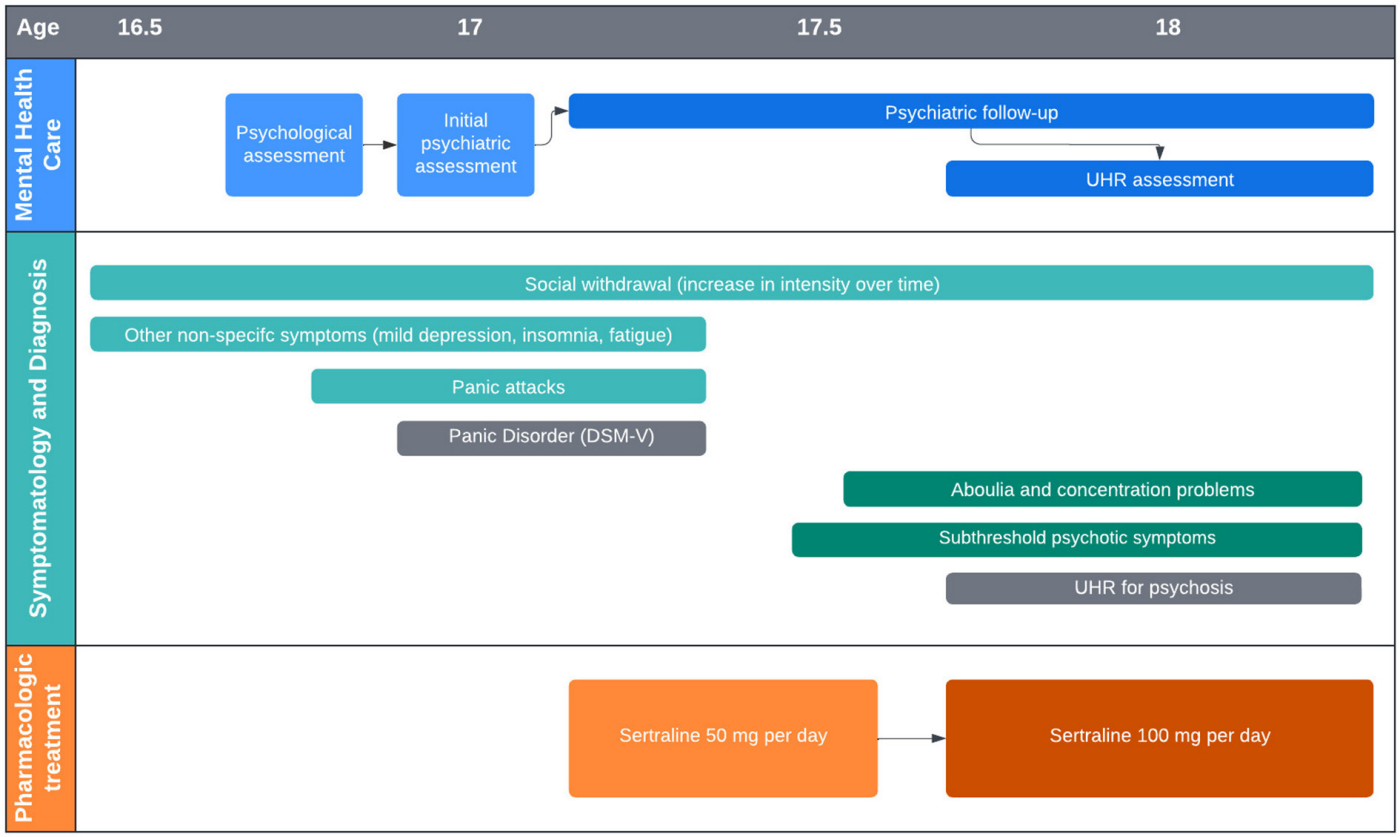
Simplified timeline of psychiatric symptoms and pharmacologic treatment.

Figure 1 shows a timeline of psychiatric symptoms and pharmacologic treatment.

## Diagnostic assessment, therapeutic intervention

Further clinical evaluation was performed and included a detailed assessment of UHR criteria for psychosis as showed in Table 2. The 16-item Version of the Prodromal Questionnaire (PQ-16), a routine screening tool for UHR of developing psychosis (17) was administered and showed a total score of 11 points (above the distress threshold of 6 or more points). The overall level of psychosocial functioning was assessed by the Social and Occupational Functioning Assessment Scale (SOFAS) (18), showing a sustained 35% drop in SOFAS score over a 12 months period. The Comprehensive Assessment of at Risk Mental States (CAARMS), was also administered. The CAARMS is a semi-structured assessment tool used by mental health professionals to identify help-seeking youth who are at UHR of psychosis and to identify the onset of the first episode of psychosis (19). Patient presented subthreshold intensity and frequency scores on non-bizarre ideas subtest and perceptual abnormalities subtest. Based on both SOFAS and CAARMS scores, the patient met the diagnostic criteria for UHR, attenuated psychosis group (APS). He did not met the diagnostic criteria for brief, limited intermittent psychotic symptoms (BLIPS), neither trait vulnerability criteria (20). Based on presenting clinical features and the clinical staging model of mental disorders, the patient was assigned a stage 1b (21).

**Table 2:** UHR for psychosis assessment results.

**Tools and subtest(s)**	**Score**	**Interpretation**	**Qualitative data**
**PQ-16**	11	Positive screening	Mild to moderate distress in all positive items
**SOFAS**	−35% (12 months)	Significant decrease	Premorbid level: 80%
**CAARMS**				
**Unusual thought content**				
Intensity	0	Not at risk	/
Frequency	0		
**Non-Bizarre ideas**				
Intensity	3	Attenuated psychosis	Suspiciousness and persecutory ideas “it seems that people look at me weirdly, that they talk about me in secret, that they could hurt me,” “this is probably an interpretation”
Frequency	4		
**Perceptual abnormalities**				
Intensity	3	Attenuated psychosis	Visual changes (distortions and/or illusions) “sometimes colors of the objects turn too intense,” “I do not understand why this is happening to me”
Frequency	4		
**Disorganized speech**				
Intensity	2	Not at risk	Subjective changes (chaotic intrusive thoughts and/or thought blockage) “sometimes my mind stops and my thoughts disappear suddenly,” “sometimes this blocks my ability to speak,” “my mind goes chaotic, there are too many thoughts at the same time”
Frequency	3		
Clinical stage	1b	UHR (APS)	/

APS, Attenuated Psychotic Syndrome; CAARMS, Comprehensive Assessment of At Risk Mental States; PQ-16, Prodromal Questionnaire, 16-item; SOFAS, Social and Occupational Functioning Assessment Scale; UHR, Ultra-High Risk for Psychosis.

In line with recent recommendations for UHR patients (8, 9), information about the diagnosis and early intervention strategies was provided and an individualized and multidisciplinary care plan was proposed. The patient still refused CBT and the follow-up included regular psychiatric monitorisation and supportive therapy, every 2–4 weeks. In order to better manage comorbid symptoms, sertraline was increased to 100 mg per day. Antipsychotic treatment was not initiated since the patient did not present severe and/ progressive UHR symptomatology (9). Both individual and family psychoeducational approaches were started even if evidence in UHR patient is still lacking and they focused on enhancing the understating of psychotic and non-psychotic symptoms, psychoeducation about the nature of anxiety and stress, engagement in treatment and increase adherence to treatment.

Over a 6 months period after the diagnosis and about a year after the onset of APS, a partial improvement of APS was observed by the clinician and the patient. The patient did not experienced a first psychotic episode over the same period.

During the initial psychiatric evaluation and the psychiatric follow-up, even if the patient was seeking care, he had a hard time in the therapeutic alliance and in explaining the symptoms, because of fear of stigmatization and of being diagnosed with a serious chronic disease. He also presented some difficulties in understanding the implications of the diagnosis and the therapeutic objectives, and in accepting the therapeutic strategies (CBT and pharmacological treatment). Both individual and family psychoeducational sessions were, according to the patient, unproductive at first, because he presented lack of motivation, but after that period, essential in order to finally understand available treatments and reduce stress and anxiety.

CARE case report guidelines were followed in the redaction of all sections the manuscript (Table 3).

**Table 3:** CARE case report guidelines.

**Topic**	**Item**	**Checklist item description**	**Reported**
Title	1	The diagnosis or intervention of primary focus followed by the words “case report”	Yes
Key words	2	2–5 key words that identify diagnoses or interventions in this case report, including “case report”	Yes
Abstract	3a	Introduction: What is unique about this case and what does it add to the scientific literature?	Yes
	3b	Main symptoms and/or important clinical findings	Yes
	3c	The main diagnoses, therapeutic interventions and outcomes	Yes
	3d	Conclusion—What is the main “take-away” lesson(s) from this case?	Yes
Introduction	4	One or two paragraphs summarizing why this case is unique	Yes
Patient information	5a	De-identified patient specific information	Yes
	5b	Primary concerns and symptoms of the patient	Yes
	5c	Medical, family, and psycho-social history including relevant genetic information	Yes
	5d	Relevant past interventions with outcomes	n/a
Clinical findings	6	Describe significant physical examination (PE) and important clinical findings	Yes
Timeline	7	Historical and current information from this episode of care organized as a timeline	Yes
Diagnostic assessment	8a	Diagnostic testing (such as PE, laboratory testing, imaging, surveys)	Yes
	8b	Diagnostic challenges (such as access to testing, financial, or cultural)	n/a
	8c	Diagnosis (including other diagnoses considered)	Yes
	8d	Prognosis (such as staging in oncology) where applicable	n/a
Therapeutic intervention	9a	Types of therapeutic intervention (such as pharmacologic, surgical, preventive, self-care)	Yes
	9b	Administration of therapeutic intervention (such as dosage, strength, duration)	Yes
	9c	Changes in therapeutic intervention (with rationale)	Yes
Follow-up and outcomes	10a	Clinician and patient-assessed outcomes (if available)	Yes
	10b	Important follow-up diagnostic and other test results	Yes
	10c	Intervention adherence and tolerability	Yes
	10d	Adverse and unanticipated events	Yes
Discussion	11a	A scientific discussion of the strengths and limitations associated with this case report	Yes
	11b	Discussion of the relevant medical literature with references	Yes
	11c	The scientific rationale for any conclusions	Yes
	11d	The primary “take-away” lessons of this case report (without references) in a one paragraph conclusion	Yes
Patient perspective	12	The patient should share their perspective in one to two paragraphs on the treatment(s) they received	Yes
Informed consent	13	Did the patient give informed consent?	Yes

n/a, non-applicable.

## Discussion

Psychiatric clinical cases are often characterized by interactions and overlaps between different diagnostic entities. Initially, this clinical case met the DSM-V criteria for panic disorder. However, the evolution of the disease has raised questions regarding a possible schizophrenia prodrome. Non-specific negative symptoms, including sleep disturbances, depressed mood, fatigue and social withdrawal were already present at the time of initial clinical presentation. Still, it was the gradual onset of positive symptoms, the attenuated psychotic symptoms, in particularly persecutory ideas and visual perceptual abnormalities, that suggested a prodromal phase of a psychotic disorder.

The DSM-V categorical approach, usually applied in clinical practice and research, is based on a list of signs and symptoms drawing a clear line between normality and psychopathology, according to a defined threshold (15). This approach is being increasingly criticized by scientific community for multiple reasons such as the excessive comorbidity between syndromes and the lack of emphasis on developmental, social, cultural and environmental context. Dimensional approaches seem to better adapt to TAY psychopathology, often characterized by early clinical presentations which include non-specific or subthreshold intensity/frequency symptoms and by the high incidence of comorbid disorders (22). In recent years, trans-diagnostic clinical staging models have gained importance, by allowing a multidimensional assessment while considering illness as a dynamic continuum from its absence to its most extreme expression (3). This broader strategy to identify at-risk patients may ultimately permit to recognize early stages of severe mental disorders, offering new management strategies tailored to patient's clinical stage, preventing the onset and/or progression of mental disorders (23). More specifically, with regard to psychotic disorders, UHR criteria represents a milestone in early detection and intervention field. The prodromal phase, previously described retrospectively, is now approached as a prospective phase. Moreover, UHR individual have a high risk to develop psychotic disorders but this pathway is neither inevitable nor the only diagnostic possibility (2). Recent scientific efforts permitted to develop clinical criteria and tools to identify UHR individuals, including the SOFAS and the CAAMS. The use of these standardized instruments can be extremely helpful to enable an early transdiagnostic approach but also to complement differential diagnosis evaluations.

More studies are needed to better identify risk and protective factors involved in transition from UHR to first episode of psychosis and schizophrenia. In this particular situation, authors questioned whether these two clinical entities are comorbid disorders or dimensional manifestations of a same disorder and whether the presence of an anxiety disorder could increase the risk of psychotic transition in UHR individuals. Prospective studies conducted in UHR individuals have found high prevalence of psychiatric comorbidities, in particular depressive (between 31 and 34%) and anxiety disorders (between 28 and 39%) (24, 25). A study on 509 UHR individuals revealed that comorbid anxiety and depressive disorders do not appear to have an effect on the risk of psychotic transition (26). Conversely, a recent study showed that, in individuals presenting psychotic experiences, non-psychotic comorbidity increases the risk of psychotic transition (27). Furthermore, in adolescents and young adults, the presence of psychotic symptoms is frequent in depressive and anxiety disorders (28).

There is still limited knowledge of the mechanisms involved in the simultaneous presence of psychotic and anxiety symptoms, including if the treatment of anxiety disorders could decrease the frequency and intensity of subthreshold psychotic symptoms and the risk of psychotic transition. Additionally, in TAY, it is particularly important to take into account developmental, social, cultural and environmental contexts. According to cohort studies, individuals who develop schizophrenia in adulthood often show developmental abnormalities in early childhood, such as speech and motor disorders and social adjustment difficulties (29). However, there are many common risk factors between psychotic and anxiety symptoms (30). Nevertheless, the knowledge of identified risk factors may provide additional clues and guide diagnostic reasoning.

Individualized and multidisciplinary assessment and management, according to the clinical stage, should be offered to the patient and the family, ideally in a specialized center. However, to date, in Brussels, Belgium, there is no service or program specifically design to UHR patients, despite scientific evidence showing the role of these structures in reducing the risk of psychotic transition and reducing the duration of untreated psychosis, compared to conventional services (31). There is, therefore, a substantial difficulty in access to current treatment strategies. Antipsychotic medication prescription to UHR patients is not recommended in clinical practice guidelines based on the current evidence. Nevertheless, pharmacological treatment of comorbidities (principally depressive and anxiety disorders) and CBT seem to decline the rate of psychotic transition (32).

In conclusion, this case report illustrates frequent difficulties on psychiatric clinical practice, particularly in transition age youth. In UHR individuals, psychiatric comorbidities, including anxiety disorders, are common and may be responsible for additional distress. The DSM-V categorical approach does not seem to adapt well to TAY psychopathology, often characterized by early clinical presentations, non-specific and/or subthreshold symptoms. A transdiagnostic and dimensional approach could better identify at-risk patients of psychiatric disorders and allow a personalized targeted-care.

## Data availability statement

The original contributions presented in the study are included in the article/supplementary material, further inquiries can be directed to the corresponding author.

## Ethics statement

Ethical review and approval was not required for the study on human participants in accordance with the local legislation and institutional requirements. Written informed consent to participate in this study was provided by the participants' legal guardian/next of kin.

## Author contributions

JR and SM contributed to the manuscript draft and research on the topic. JR was responsible for the psychiatric assessment and follow-up of the patient. HN provided clinical advice to JR. HN and VD reviewed the case report and article as senior authors. All authors approved the submitted version.

## Funding

This study is part of the University Chair Psychiatry in Transition in a World in Transition (Université Libre de Bruxelles, Brussels, Belgium), supported by the Julie Renson Fund, the Queen Fabiola Fund and the King Baudouin Foundation. Apart from the financial contribution in research activities, the funding institutions have no role in data collection, diagnostic assessment, or therapeutic strategies.

## Conflict of interest

The authors declare that the research was conducted in the absence of any commercial or financial relationships that could be construed as a potential conflict of interest.

## Publisher's note

All claims expressed in this article are solely those of the authors and do not necessarily represent those of their affiliated organizations, or those of the publisher, the editors and the reviewers. Any product that may be evaluated in this article, or claim that may be made by its manufacturer, is not guaranteed or endorsed by the publisher.

## References

[1] Solmi M Radua J Olivola M Croce E Soardo L Salazar de Pablo G. Age at onset of mental disorders worldwide: large-scale meta-analysis of 192 epidemiological studies. Mol Psychiatry. (2022) 27:281–95. 10.1038/s41380-021-01161-7 34079068 PMC8960395

[2] McGorry PD Hickie IB. Clinical Staging in Psychiatry. Cambrigde: Cambridge University Press (2019).

[3] Shah JL Scott J McGorry PD Shane PMC Matcheri SK Barnaby N. Transdiagnostic clinical staging in youth mental health: a first international consensus statement. World Psychiatry. (2020) 19:233–42. 10.1002/wps.20745 32394576 PMC7215079

[4] Hafner H Maurer K Loffler W Fatkenheuer B an der Heiden W Riecher-Rossler A. The epidemiology of early schizophrenia Influence of age and gender on onset and early course. Br J Psychiatry. (1994) 23:29–38. 10.1192/S0007125000292714 8037899

[5] Pallanti S Cantisani A Grassi G. Anxiety as a core aspect of schizophrenia. Curr Psychiatry. (2013) 15:1–5. 10.1007/s11920-013-0354-7 23532444

[6] Achim AM Maziade M Raymond E Oliver D Mérette C Roy M-A. How prevalent are anxiety disorders in schizophrenia? A meta-analysis and critical review on a significant association. Schizophr Bull. (2011) 37:811–21. 10.1093/schbul/sbp148 19959704 PMC3122284

[7] McGorry PD Nelson B Amminger GP Bechdolf A Francey SN Berger G. Intervention in individuals at ultra-high risk for psychosis: a review and future directions. Clin Psychiatry. (2009) 70:1206–12. 10.4088/JCP.08r04472 19573499

[8] Orygen. The National Centre of Excellence in Youth Mental Health. Australian Clinical Guidelines for Early Psychosis (2nd edition update). Melbourne, VIC: Orygen (2016).

[9] Schmidt SJ Schultze-Lutter F Schimmelmann BG Marit NP Salokangas RKR Riecher-Rossler A. EPA guidance on the early intervention in clinical high risk states of psychoses. Eur Psychiatry. (2015) 30:388–404. 10.1016/j.eurpsy.2015.01.013 25749390

[10] Wechsler D. Wechsler Adult Intelligence Scale, Fourth Edition (WAIS-IV). San Antonio, TX: Pearson (2008).

[11] Beck AT Steer RA Brown GK. The Beck Depression Inventory Second Edition (BDI-II). San Antonio, TX: Psychological Corporation (1996).

[12] Osman A Kopper BA Barrios F Gutierrez PM Bagge CL. Reliability and validity of the Beck depression inventory-II with adolescent psychiatric inpatients. Psychol Assess. (2004) 16:120–32. 10.1037/1040-3590.16.2.120 15222808

[13] Shear MK Brown TA Barlow DH Money R Sholomskas S Woods SW. Multicenter collaborative panic disorder severity scale. Am J Psychiatry. (1997) 154:1571–5. 10.1176/ajp.154.11.1571 9356566

[14] Furukawa TA Shear MK Barlow DH Gorman JM Woods SW Money R. Evidence-based guidelines for interpretation of the Panic Disorder Severity Scale Depress. Anxiety. (2009) 26:922–9. 10.1002/da.20532 19006198 PMC2760657

[15] American Psychiatric Association. Diagnostic and Statistical Manual of Mental Disorders. 5th ed. Washington, DC: American Psychiatric Association (2013).

[16] Walter HJ Bukstein OG Abright AR Keable H Ramtekkar U Ripperger-Suhler J. Clinical practive guideline for the assessment and treatment of children and adolescents with anxiety disorders. J Am Acad Child Adolesc Psychiatry. (2020) 59:1107–24. 10.1016/j.jaac.2020.05.005 32439401

[17] Ising HK Veling W Loewy RL Rietveld MW Rietjijk J Dragt S. The validity of the 16-item version of the Prodromal Questionnaire (PQ-16) to screen for ultra high risk of developing psychosis in the general help-seeking population. Schizophr Bull. (2012) 38:1288–96. 10.1093/schbul/sbs068 22516147 PMC3713086

[18] Rybarczyk B. Social and occupational functioning assessment scale (SOFAS). In: Kreutzer JS DeLuca J Caplan B. Encyclopedia of Clinical Neuropsychology. New York, NY: Springer (2011).

[19] Yung AR Yuen HP McGorry PD Phillips LJ Kelly D Dell'Olio M. Mapping the onset of psychosis: the Comprehensive Assessment of At-Risk Mental States. Aust N Z J Psychiatry. (2005) 39:964–71. 10.1080/j.1440-1614.2005.01714.x 16343296

[20] Yung AR Phillips LJ Yuen HP McGorry P. Risk factors for psychosis in an ultra high-risk group: psychopathology and clinical features. Schizophr Res. (2004) 1:131–42. 10.1016/S0920-9964(03)00192-0 14984872

[21] McGorry P Keshavan M Goldstone S Amminger P Allot K Berk M. Biomarkers and clinical staging in psychiatry. World Psychiatry. (2014) 13:211–23. 10.1002/wps.20144 25273285 PMC4219053

[22] Cuthbert BN. The RDoC framework: facilitating transition from ICD/DSM to dimensional approaches that integrate neuroscience and psychopathology. World Psychiatry. (2014) 13:28–35. 10.1002/wps.20087 24497240 PMC3918011

[23] McGorry PD Hartmann JA Spooner R Barnaby N. Beyond the “at risk mental state” concept: transitioning to transdiagnostic psychiatry. World Psychiatry. (2018) 17:133–42. 10.1002/wps.20514 29856558 PMC5980504

[24] Salokangas RK Ruhrmann S Von Reventlow HG Heinimaa M Svirskis T From T. Axis I diagnoses and transition to psychosis in clinical high-risk patients EPOS project: prospective follow-up of 245 clinical high-risk outpatients in four countries. Schizophr Res. (2021) 138:192–7. 10.1016/j.schres.2012.03.008 22464922

[25] Lim J Rekhi G Rapisarda A Lam M Kraus M Keefe R. Impact of psychiatric comorbidity in individuals at Ultra High Risk of psychosis - Findings from the Longitudinal Youth at Risk Study (LYRIKS). Schizophr Res. (2015) 164:8–14. 10.1016/j.schres.2015.03.007 25818728

[26] Fusar-Poli P Nelson B Valmaggia L Yung AR McGuire PK. Comorbid depressive and anxiety disorders in 509 individuals with an at-risk mental state: impact on psychopathology and transition to psychosis. Schizophr Bull. (2014) 40:120–31. 10.1093/schbul/sbs136 23180756 PMC3885287

[27] Hasmi L Pries LK Ten Have M de Graaf R van Dorsselaer S Bak M. What makes the psychosis 'clinical high risk' state risky: psychosis itself or the co-presence of a non-psychotic disorder? Epidemiol Psychiatr Sci. (2021) 30:1–10. 10.1017/S204579602100041X 34225831 PMC8264801

[28] Wigman JT Van Nierop M Vollebergh WA Lieb R Beesdo-Baum K Wittchen HU. Evidence that psychotic symptoms are prevalent in disorders of anxiety and depression, impacting on illness onset, risk, and severity—implications for diagnosis and ultra-high risk research. Schizophr Bull. (2012) 38:247–57. 10.1093/schbul/sbr196 22258882 PMC3283146

[29] Millan MJ Andrieux A Bartzokis G Cadenhead K Dazzan P Fusar-Poli P Altering the course of schizophrenia: progress and perspectives. Nat Rev Drug Discov. (2016) 15:485–515. 10.1038/nrd.2016.28 26939910

[30] Hall J. Schizophrenia – an anxiety disorder? Br J Psychiatry. (2017) 211:262–3. 10.1192/bjp.bp.116.195370 29092833

[31] National Collaborating Centre for Mental Health. Psychosis and Schizophrenia in Adults: Treatment and Management. London: NICE Clinical Guideline 178 (2014).

[32] Formica MJC Phillips LJ Hartmann JA Yung AR Wood SJ Lin A. Has improved treatment contributed to the declining rate of transition to psychosis in ultra-high-risk cohorts? Schizophr Res. (2022) 243:276–84. 10.1016/j.schres.2020.04.028 32402606

